# Electrophysiological evidence for the characteristics of implicit self-schema and other-schema in patients with major depressive disorder: An event-related potential study

**DOI:** 10.3389/fpsyt.2023.1131275

**Published:** 2023-04-11

**Authors:** Jia-yu Yao, Zi-wei Zheng, Yi Zhang, Shan-shan Su, Yuan Wang, Jing Tao, Yi-hua Peng, Yan-ru Wu, Wen-hui Jiang, Jian-yin Qiu

**Affiliations:** Shanghai Mental Health Center, Shanghai Jiao Tong University School of Medicine, Shanghai, China

**Keywords:** major depressive disorder (MDD), implicit schemas, self-schema, other-schema, event-related potential

## Abstract

**Background:**

The significance of implicit self-schema and other-schema in major depressive disorder (MDD) is highlighted by both cognitive theory and attachment theory. The purpose of the current study was to investigate the behavioral and event-related potential (ERP) characteristics of implicit schemas in MDD patients.

**Methods:**

The current study recruited 40 patients with MDD and 33 healthy controls (HCs). The participants were screened for mental disorders using the Mini-International Neuropsychiatric Interview. Hamilton Depression Rating Scale-17 and Hamilton Anxiety Rating Scale-14 were employed to assess the clinical symptoms. Extrinsic Affective Simon Task (EAST) was conducted to measure the characteristics of implicit schemas. Meanwhile, reaction time and electroencephalogram data were recorded.

**Results:**

Behavioral indexes showed that HCs responded faster to positive self and positive others than negative self (*t* = −3.304, *p* = 0.002, Cohen’s *d* = 0.575) and negative others (*t* = −3.155, *p* = 0.003, Cohen’s *d* = 0.549), respectively. However, MDD did not show this pattern (*p* > 0.05). The difference in other-EAST effect between HCs and MDD was significant (*t* = 2.937, *p* = 0.004, Cohen’s *d* = 0.691). The ERP indicators of self-schema showed that under the condition of positive self, the mean amplitude of LPP in MDD was significantly smaller than that in HCs (*t* = −2.180, *p* = 0.034, Cohen’s *d* = 0.902). The ERP indexes of other-schema showed that HCs had a larger absolute value of N200 peak amplitude for negative others (*t* = 2.950, *p* = 0.005, Cohen’s *d* = 0.584) and a larger P300 peak amplitude for positive others (*t* = 2.185, *p* = 0.033, Cohen’s *d* = 0.433). The above patterns were not shown in MDD (*p* > 0.05). The comparison between groups found that under the condition of negative others, the absolute value of N200 peak amplitude in HCs was larger than that in MDD (*t* = 2.833, *p* = 0.006, Cohen’s *d* = 1.404); under the condition of positive others, the P300 peak amplitude (*t* = −2.906, *p* = 0.005, Cohen’s *d* = 1.602) and LPP amplitude (*t* = −2.367, *p* = 0.022, Cohen’s *d* = 1.100) in MDD were smaller than that in HCs.

**Conclusion:**

Patients with MDD lack positive self-schema and positive other-schema. Implicit other-schema might be related to abnormalities in both the early automatic processing stage and the late elaborate processing stage, while the implicit self-schema might be related only to the abnormality in the late elaborate processing stage.

## 1. Introduction

Major depressive disorder (MDD), a common mood disorder with approximately 350 million patients worldwide (1), has become an important global public health problem with high incidence, relapse, and suicide rates. Symptoms such as self-denial, self-deprecation, interpersonal avoidance, and high interpersonal sensitivity are key features in depression (2).

The abnormalities of implicit schemas are considered by cognitive theory and attachment theory to be the core issues of MDD. Schemas refer to the internal cognitive structure based on which individuals select, process, and organize information. Schemas are usually implicit and are activated in response to stressful events, especially interpersonal ones (3). According to Beck (4), MDD patients are characterized by negative views of self, others, and the world. Among them, the views of self-include representations and beliefs about the past, present, and future associated with oneself, i.e., self-schema, also known as self-representation. The belief about others is other-schema, also known as other-representation. Bowlby’s attachment theory emphasizes that in MDD patients, the other-schema that plays a central role is the representation of those with whom the individual has intimate relationships, such as parents and partners (5).

Most previous studies explored the characteristic of explicit self-schema in MDD through Self-Referential Encoding Task (SRET). They found that MDD patients perceived negative words as more appropriate for describing themselves, whereas healthy controls (HCs) perceived the opposite (6–8). However, since SRET asks subjects to judge whether the adjectives presented describe themselves, it is vulnerable to expectancy effects. Furthermore, because the explicit and implicit self-schemas are incongruent in MDD patients (9), some studies have investigated the implicit self-schema through Implicit Association Test (IAT) and Go/No-go Association Task (GNAT) with reaction time (RT) as the main behavioral index. Nevertheless, the findings remain controversial. For example, both Risch et al. (10) and Romero et al. (11) found that MDD had a more negative implicit self-schema than HCs, while others found different results (12–15). Moreover, only a few researchers have investigated the other-schema in MDD patents. However, both cognitive theory and attachment theory emphasize the significance of both self-schema and other-schema in MDD, especially the other-schema toward parents and partners, making it essential to explore the characteristic of the other-schema in this population. To the best of our knowledge, only the study conducted by Yao et al. (16) revealed the presence of positive self-schema and other-schema in HCs, while MDD patients lacked positive self-schema and had negative other-schema of parents and partners. In their study, External Affect Simon Task (EAST) was conducted, which offered an advantage over other paradigms since it can simultaneously measure both self-schema and other-schema (17). However, they did not explore the detailed cognitive processes since only RT was analyzed.

Event-Related Potential (ERP) has a higher temporal resolution than behavioral indexes, allowing for a more detailed exploration of cognitive processes. N200, P300, and late positive potential (LPP) are ERP components associated with neural activity patterns of implicit schemas, though the findings varied widely. N200 is a negative wave with a frontal scalp distribution that peaks around 250–350 ms after stimulus onset (18) and is associated with conflict monitoring and response inhibition (19). A study by Wu et al. (20) on healthy college students suggested that when negative words were paired with self-words, subjects showed a greater absolute value of N200 peak, suggesting that the pairing of negative words and self-words was inconsistent with subjects’ implicit attitudes, i.e., the presence of a positive self-schema in HCs. However, similar results were not found in another study (21). P300 is a positive wave that peaks around 300–400 ms after stimulus onset over parietal sites and is related to the allocation of attention and cognitive resources (19). Some studies consider LPP and P300 as the same component, thus referring to the wave appearing at 300–400 ms or before 600 ms as early LPP (15, 22), and the wave appearing after 600 ms over the centro-parietal scalp as late LPP (15, 19, 23). Late LPP is related to the degree of arousal and delicate processing of stimuli (8, 24). Both Auerbach et al. (6) and Dainer-Best et al. (25) combined P300, LPP and SRET, but found different results. The former study found that MDD showed greater P300 and LPP amplitudes in the negative self condition, while HCs showed the opposite pattern (6). This suggested that MDD patients not only assigned more attention to the negative self, but also processed the negative self in more detail. However, in the study by Dainer-Best et al. (25), no significant results for LPP amplitudes were found. Notably, Auerbach et al.’s (6) study only included female adolescent MDD patients, and MDD sample in Dainer-Best et al.’s (25) study was not clinically diagnosed, but assessed only by telephone interview. Additionally, Grundy et al. (26) found that subjects with low self-esteem had a greater P300 amplitude in the positive self condition. Given that MDD patients is often accompanied by low self-esteem, further clarification of the neural activity pattern of the self-schema is needed. Furthermore, although several studies have involved the neural activity of other-schema, they have focused on HCs and have not yielded consistent conclusions. For example, Chen et al. (27) found that HCs had a greater P300 amplitude in the positive others condition than in the negative others condition, whereas Wu et al. (20) found the opposite results.

Therefore, the current study aimed to explore the neural activity patterns of both implicit self-schema and other-schema in MDD by combining EAST and ERP. For behavioral indexes, it was hypothesized that HCs responded to positive self-words and positive other-words more quickly. For ERP components, we hypothesized that in HCs, the absolute value of N200 peak amplitude was greater in the negative self condition and negative others condition, whereas the P300 amplitudes and the LPP amplitudes were greater in the negative self and negative others condition. However, MDD would show reduced or reversed patterns compared to HCs.

## 2. Materials and methods

### 2.1. Participants

All subjects with MDD were recruited from outpatients of Shanghai Mental Health Center from September 2021 to October 2022. All enrolled patients were evaluated with the following inclusion and exclusion criteria. (1) Inclusion criteria: (a) meeting the diagnosis of MDD in the Diagnostic and Statistical Manual of Mental Disorders (DSM), Fifth Edition; (b) currently in a depressive episode; (c) scoring ≥ 17 on the 17-item Hamilton Depression Rating Scale (HAMD-17) and scoring ≤ 21 on the 14-item Hamilton Anxiety Rating Scale (HAMA-14); (d) meeting the diagnosis of depression in the Mini-International Neuropsychiatric Interview (MINI) and without any psychotic symptoms; (e) at least a junior high school education level; (f)18–55 years old; (g) not receiving medication and systematic psychotherapy in the last 6 months; (h) with sufficient audiovisual level to complete the study. (2) Exclusion criteria: (a) currently presence of serious physical disease; (b) history of brain injury; (c) meeting the diagnosis of other mental disorders in MINI; (d) presence of serious suicide attempts; (e) inability to complete the study due to other problems such as mental retardation.

All HCs were age-matched and gender-matched adults recruited from the community through advertisement from May 2022 to August 2022. All enrolled HCs were evaluated with the following inclusion and exclusion criteria. (1) Inclusion criteria: (a) no history of any mental disorders; (b) no history of any mental disorders across three family generations; (c) scoring <7 on both HAMD-17 and HAMA-14; (d) at least a junior high school education level; (e) 18–55 years old; (f) with sufficient audiovisual level to complete the study; (2) Exclusion criteria: (a) currently presence of serious physical disease; (b) history of brain injury.

According to previous studies (6), a medium effect size was assumed in the current study, which required a minimum of 50 participants (25 in each group) (28). 40 MDD patients and 33 HCs met the corresponding inclusion and exclusion criteria and were included in the current study.

This study has been approved by the Ethics Committee of Shanghai Mental Health Center (2021ky-122). All participants have signed the informed consent.

### 2.2. Measures and procedure

All subjects were interviewed with MINI, HAMD-17, and HAMA-14 to assess their clinical symptoms and determine whether the inclusion criteria were met. Among them, MINI was designed to screen for mental disorders based on DSM-4. HAMD-14 was used to access anxiety symptoms, with higher scores indicating more severe anxiety symptoms. HAMD-17 was employed to access depressive symptoms including low mood, weight loss, somatic symptoms and so on. A HAMD-17 score of less than 7 was classified as no depressive symptoms, 7–16 as mild, 17–24 as moderate and more than 24 as severe.

**FIGURE 1:**
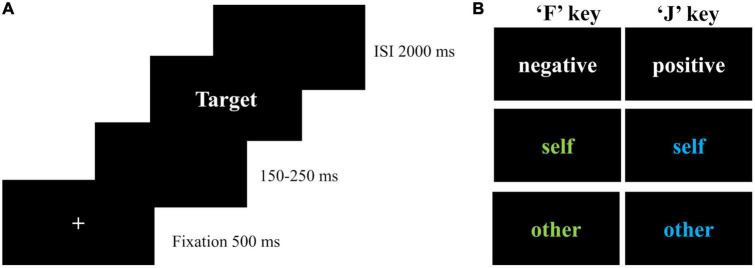
Extrinsic affective Simon task. **(A)** Flowchart of each trial; **(B)** conditions in EAST; ISI, inter-stimulus interval; self-words included I, myself, me, and self (“我”, “自己”, “本人”, “自我” in Chinese). Other-words included dad, mom, partner, and lover (“爸爸”, “妈妈”, “伴侣”, “恋人” in Chinese). Positive words included nice, warm, lovely, kind, excellent, and capable (“美好的”, “温暖的”, “可爱的”, “善良的”, “优秀的”, “能干的”). Negative words included terrible, incompetent, useless, evil, lame, and disgusting (“糟糕的”, “无能的”, “没用的”, “罪恶的”, “差劲的”, “讨厌的”).

Based on IAT, EAST was developed by De Houwer (17), which can measure self-schema and other-schema at the same time. Participants were asked to press the “F” or “J” keys according to the valence of attribute words or the color of object words presented on the screen. Consistent with a previous study (16), attribute words were printed in white, including positive words and negative words. Object words were printed in blue or green, including self-words and other-words. For each trial, a fixation was presented in the center of the screen for 500 ms. To avoid the influence of subjects’ anticipation of the upcoming word on ERP components, a blank screen with a duration of 150–250 ms appeared before the stimulus onset. After the stimulus was onset, subjects were asked to respond as quickly as possible. Once they pressed the “F” or “J” key, an inter-stimulus interval was presented for 2,000 ms to avoid the effect of the next trial on the late ERP components of the previous trial (Figure 1).

There were two practice blocks and six formal blocks in EAST. The first block consisted of eight positive words and eight negative words. Participants were asked to press the “F” key for negative words and the “J” key for positive words, assigning negative and positive attributes to the “F” and “J” keys, respectively. The second practice block consisted of four self-words and four other-words, each repeated twice in blue and green. In this block, participants were asked to press the “F” key for words in green, and the “J” key for words in blue, assigning negative attributes to green and positive attributes to blue. The formal blocks included six conditions (blue self-words, blue other-words, green self-words, green other-words, white positive words, and white negative words). To ensure that there were enough trials for ERP analysis, the number of trials in the formal blocks was increased from 144 trials in the original task (16) to 360 trials, with 60 trials in each block presented with randomization. The instruction of formal blocks combined the instructions of the two practice blocks, with responses based on valence if the words were white, or based on colors if the words were blue or green.

To avoid fatigue effects, there was a 30-s rest period between two blocks. The task was programmed by E-Prime 3.0, through which RT and accuracy were recorded.

### 2.3. Electroencephalography (EEG) data recordings and preprocessing

EEG data were acquired during EAST using ANT Neuro system with 64 scalp sites. The sampling rate was 500 Hz, and the impedance of each electrode was below 10 kΩ. The online reference electrode was CPz, and the offline reference electrodes were M1 and M2.

EEGLAB toolbox in Matlab 2013b was used to preprocess the EEG data. There were seven steps of preprocessing. (1) locating the channels with international standard 10-20 system; (2) filtering the data with a bandpass in the range of 0.1–30 Hz; (3) segmenting the data in the range of 200 ms before stimulus onset to 1,000 ms after post-stimulus and epochs with incorrect answers were removed; (4) the data of 200 ms pre-stimulus were used for baseline correction; (5) re-referencing the data to M1 and M2 electrodes; (6) independent component analysis was used to remove artifacts such as eye movements; and (7) removing epochs with amplitudes at any electrode sites exceeding ± 80 μv.

### 2.4. Statistical analyses

To compare the demographic and clinical differences between MDD patients and HCs, independent *t*-test was used for continuous variables such as age, HAMD-17 and HAMA-14. For categorical variables (i.e., gender and education level), chi-square test or Fisher’s exact test was employed. To ensure the comparability of our results with previous studies, the method of processing RT outliers was consistent with previous studies (16, 17). Specifically, RTs below 300 ms and RTs above 3,000 ms were taken as 300 ms and 3,000 ms, respectively. The average proportion of this kind of trials was 0.23% across all participants and conditions, with a range of 0–1.68%. There were six indexes in EAST, four of which were the average RTs in the four conditions (i.e., positive self, negative self, positive others and negative others). The other two indexes were the self-EAST effect (RT for negative self condition minus RT for positive self condition) and the other-EAST effect (RT for positive others condition minus RT for negative others condition). A higher EAST effect indicated a more positive self-schema or other-schema. As we aimed to focus on the characteristics of self-schema and other-schema separately, rather than on their relationships, 2 (valence: positive/negative) * 2 (group: MDD/HC) repeated measures analysis of variances (RMANOVAs) were performed on RT for self-words and other-words, respectively. To compare the differences in self-EAST effect and other-EAST effect between groups, an independent sample *t*-test was employed with Bonferroni correction. SPSS 22.0 was used to perform the above analysis.

After visual inspection of the grand averaged waveforms and topographic maps of the current study, along with previous studies (15, 18, 19, 23), we analyzed three ERP components with the following time windows and electrodes. (1) The peak amplitude and latency of N200 (250–350 ms) were calculated across Fz, F1, F2, F3, and F4. (2) The peak amplitude and latency of P300 (300–400 ms) were calculated across Pz, P1, P2, P3, P4, CP1, CP2, CP3, and CP4. (3) The mean amplitude of LPP (600–1,000 ms) was calculated across Cz, C1, C2, C3, and C4. Similar to the analysis of RT, 2 (valence: positive/negative) × 2 (group: MDD/HCs) RMANOVAs were performed for self-words and other-words, respectively. Greenhouse–Geisser correction was conducted if the sphericity test was violated. The significant level was 0.05. R 4.0.2 with “bruceR” package was used to perform the above analysis.

## 3. Results

### 3.1. Demographic and clinical characteristics

A total of 40 MDD patients and 33 HCs were included in the study. No significant differences were observed in age (*t* = −0.604, *p* = 0.106), gender (*X*^2^ = 0.031, *p* = 0.861), and education level (*p* = 0.644). Among them, chi-square test was conducted on gender and Fisher’s exact test was conducted on education level given that 50% cells had an expected count less than 5. The scores of MDD patients were significantly higher than those of HCs in HAMD-17 (*t* = −32.035, *p* < 0.001), and HAMA-14 (*t* = −18.928, *p* < 0.001) (Table 1).

**TABLE 1:** Demographic and clinical characteristics of MDD and HCs.

	HCs (*n* = 33)	MDD (*n* = 40)	*X*^2^/*t*	*p*
Age (M ± SD)	28.15 ± 9.11	29.28 ± 6.76	−0.604	0.106
Gender (*n*)			0.031	0.861
Male	8	9		
Female	25	31		
Education level (*n*)			/	0.644
Middle school	1	3		
College	32	37		
HAMD-17 (M ± SD)	2.21 ± 1.65	20.90 ± 3.21	−32.035	<0.001
HAMA-14 (M ± SD)	1.06 ± 1.37	15.08 ± 4.43	−18.928	<0.001

MDD, major depressive disorder; HCs, healthy controls; M, mean; SD, standard deviation.

### 3.2. Behavioral outcomes

**FIGURE 2:**
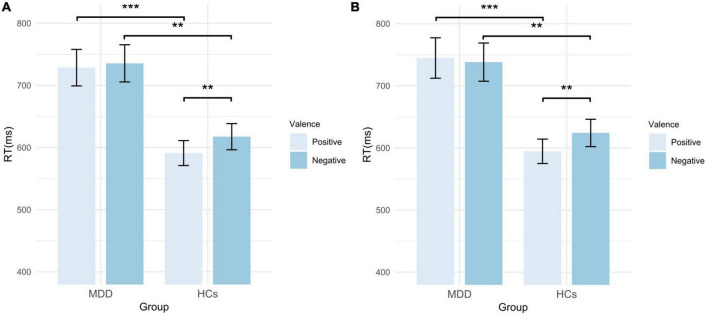
RTs of MDD patients and HCs in EAST. **(A)** RTs of self-words; **(B)** RTs of other-words. MDD, major depressive disorder; HCs, healthy controls; RT, reaction time; EAST, External Affect Simon Task; error bars represent the standard error. **p < 0.01; ***p < 0.001.

Table 2 shows the RTs of MDD patients and HCs in different conditions. For self-schema, the main effect of group (*F* = 11.905, *p* < 0.001, η^2^p = 0.144) and valence (*F* = 6.335, *p* = 0.014, η^2^p = 0.082) were significant, but the interaction effect (*F* = 2.147, *p* = 0.147, η^2^p = 0.029) was not significant. However, the exploratory analysis found that HCs responded significantly faster to the positive self-words than to the negative self-words (*t* = −2.690, *p* = 0.009, Cohen’s *d* = 0.472), whereas no difference was found between negative self-words and positive self-words in MDD patients (*t* = −0.782, *p* = 0.437, Cohen’s *d* = 0.125). In addition, HCs responded faster to both positive self-words (*t* = 3.700, *p* < 0.001, Cohen’s *d* = 2.458) and negative self-words (*t* = 3.101, *p* = 0.003, Cohen’s *d* = 2.111) than MDD patients. For other-schema, the main effect of group (*F* = 11.339, *p* = 0.001, η^2^p = 0.138) and the interaction effect (*F* = 8.625, *p* = 0.004, η^2^p = 0.108) were significant, while the main effect of valence (*F* = 3.460, *p* = 0.067, η^2^p = 0.046) was not significant. *Post hoc* analysis found that the RT for positive other-words was significantly greater than that for negative other-words in HCs (*t* = −3.240, *p* = 0.002, Cohen’s *d* = 0.568). And still, no difference was found between negative other-words and positive other-words in MDD patients (*t* = 0.801, *p* = 0.426, Cohen’s *d* = −0.127). Moreover, HCs responded faster to both positive other-words (*t* = 3.750, *p* < 0.001, Cohen’s *d* = 2.882) and negative other-words (*t* = 2.895, *p* = 0.005, Cohen’s *d* = 2.186) than MDD patients (Figure 2). Independent sample *t*-test was performed on self-EAST effect and other-EAST effect. Though both the self-EAST effect (HCs: 29.36 ± 45.83; MDD: 6.96 ± 63.61) and other-EAST effect (HCs: 29.60 ± 53.89; MDD: −6.64 ± 51.30) of HCs were greater than that of MDD, only the difference of other-EAST effect was significant (other-EAST effect: *t* = 2.937, *p* = 0.008, Cohen’s *d* = 0.691; self-EAST effect: *t* = 1.465, *p* = 0.294, Cohen’s *d* = 0.398) (Figure 2).

**TABLE 2:** RTs of MDD and HCs in EAST.

		Positive		Negative	
		**M ± SD**	**95% CI of M**	**M ± SD**	**95% CI of M**
HCs	Self-words	591.11 ± 115.42	[550.184, 632.037]	617.47 ± 120.64	[574.694, 660.251]
	Other-words	594.54 ± 113.09	[554.439, 634.637]	624.14 ± 126.39	[579.326, 668.955]
MDD	Self-words	728.54 ± 185.72	[669.142, 787.936]	735.50 ± 189.05	[675.041, 795.962]
	Other-words	744.73 ± 205.69	[678.951, 810.517]	738.09 ± 194.62	[675.848, 800.331]

MDD, major depressive disorder; HCs, healthy controls; RT, reaction time; EAST, External Affect Simon Task; M, mean; SD, standard deviation; CI, confidence interval.

### 3.3. ERP outcomes

Six MDD patients and seven HCs were excluded due to excessive artifacts in more than half of their trials. The EEG data of four MDD patients were not acquired due to equipment problems. Thus, twenty-six HCs and thirty MDD patients were included in the analysis of ERP outcomes. To clarify whether the results of demographic characteristics and behavioral indicators were consistent for the complete sample and the sample included in the ERP analysis, the above statistical analyses were conducted again on the sample included in the ERP analysis. The results were presented in the Supplementary material and were consistent with the results of the complete sample described above. For ERP outcomes, RMANOVA was performed for self-words and other-words, respectively.

#### 3.3.1. Comparison of the ERP differences of self-words between MDD and HCs

**FIGURE 3:**
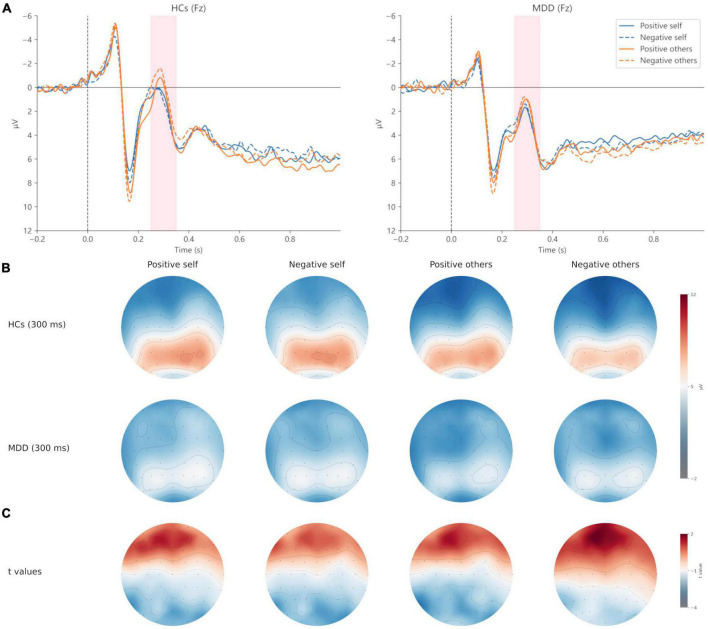
Grand averages **(A)** of N200 (250–350 ms) at Fz electrodes under positive self (blue solid lines), negative self (blue dashed lines), positive others (orange solid lines), and negative others (orange dashed lines) condition. Topographic maps of N200 at 300 ms **(B)** under four conditions. Topographic distribution of t values for N200 between MDD and HCs **(C)** under four conditions. MDD, major depressive disorder; HCs, healthy controls.

**FIGURE 4:**
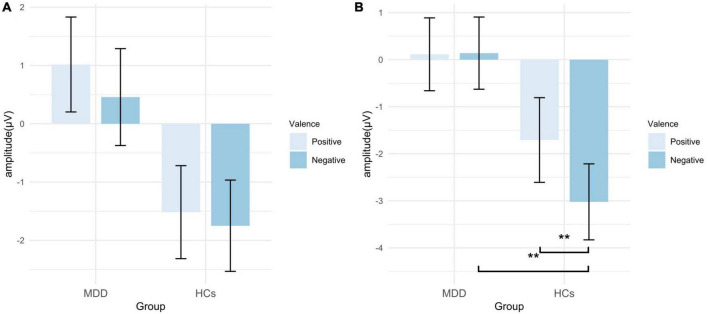
Peak amplitude of N200 in EAST. **(A)** Amplitudes of self-words; **(B)** amplitudes of other-words. MDD, major depressive disorder; HCs, healthy controls; EAST, external affect Simon task; error bars represent the standard error. **p < 0.01.

For the peak amplitude of N200, the main effect of valence (*F* = 1.311, *p* = 0.257, η^2^p = 0.024) and interaction effect (*F* = 0.222, *p* = 0.640, η^2^p = 0.004) were not significant, but the main effect of group was significant (*F* = 4.670, *p* = 0.035, η^2^p = 0.080). Regarding the latency of N200, the main effect of valence (*F* = 3.045, *p* = 0.087, η^2^p = 0.053) and group (*F* = 1.754, *p* = 0.191, η^2^p = 0.031) was not significant, whereas the interaction was significant (*F* = 4.420, *p* = 0.040, η^2^p = 0.076). *Post hoc* analysis showed that in HCs, the latency of positive self-words was significantly greater than that of negative self-words (*t* = 2.628, *p* = 0.011, Cohen’s *d* = 0.520), but no significant difference was found in MDD (*t* = −0.262, *p* = 0.794, Cohen’s *d* = 0.048). Additionally, the latency of negative self-words in MDD was greater than that in HCs (*t* = 2.211, *p* = 0.0.031, Cohen’s *d* = 0.604), and no group difference was found in the positive self conditions (*t* = 0.126, *p* = 0.900, Cohen’s *d* = 0.035) (Figures 3, 4; Table 3).

**TABLE 3:** The N200, P300, and LPP of MDD and HCs in EAST (M ± SD).

			Positive		Negative	
			**Latency (ms)**	**Peak/Mean amplitude (μV)**	**Latency (ms)**	**Peak/Mean amplitude (μV)**
N200	HCs	Self-words	284.92 ± 28.84	−1.52 ± 4.07	272.00 ± 26.30	−1.75 ± 3.99
		Other-words	283.23 ± 25.07	−1.71 ± 4.59	278.15 ± 24.46	−3.02 ± 4.12
	MDD	Self-words	285.80 ± 23.20	1.02 ± 4.46	287.00 ± 24.44	0.46 ± 4.55
		Other-words	290.00 ± 18.20	0.11 ± 4.24	288.67 ± 21.52	0.14 ± 4.20
P300	HCs	Self-words	320.92 ± 38.33	6.53 ± 4.45	321.31 ± 38.78	6.48 ± 4.59
		Other-words	317.46 ± 33.16	6.21 ± 4.27	314.46 ± 32.16	5.27 ± 4.68
	MDD	Self-words	330.93 ± 41.79	3.53 ± 4.70	331.13 ± 42.36	3.49 ± 4.42
		Other-words	321.73 ± 37.65	2.74 ± 4.60	320.60 ± 37.48	3.11 ± 4.64
LPP	HCs	Self-words	/	5.69 ± 3.42	/	5.65 ± 3.46
		Other-words	/	6.36 ± 3.21	/	6.00 ± 3.31
	MDD	Self-words	/	3.79 ± 3.09	/	4.06 ± 3.72
		Other-words	/	4.20 ± 3.56	/	4.46 ± 3.64

MDD, major depressive disorder; HCs, healthy controls; M, mean; SD, standard deviation.

**FIGURE 5:**
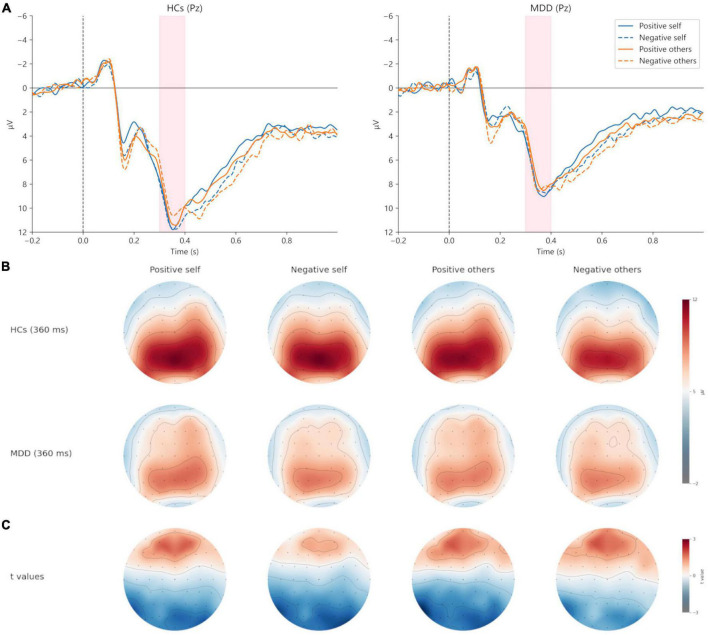
Grand averages **(A)** of P300 (300–400 ms) at Pz electrodes under positive self (blue solid lines), negative self (blue dashed lines), positive others (orange solid lines), and negative others (orange dashed lines) condition. Topographic maps of P300 at 360 ms **(B)** under four conditions. Topographic distribution of t values for P300 between MDD and HCs **(C)** under four conditions. MDD, major depressive disorder; HCs, healthy controls.

**FIGURE 6:**
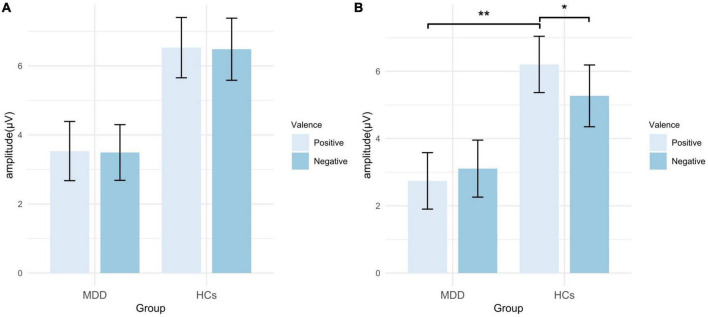
Peak amplitude of P300 in EAST. **(A)** Amplitudes of self-words; **(B)** amplitudes of other-words. MDD, major depressive disorder; HCs, healthy controls; EAST, external affect Simon task; error bars represent the standard error. *p < 0.05; **p < 0.01.

For the peak amplitude of P300, the main effect of valence (*F* = 0.026, *p* = 0.873, η^2^p = 0.000) and interaction (*F* = 0.000, *p* = 0.993, η^2^p = 0.000) were not significant, but the main effect of group was significant (*F* = 6.369, *p* = 0.015, η^2^p = 0.105). In terms of the latency of P300, no significant results were found (*p* > 0.05) (Figures 5, 6; Table 3).

**FIGURE 7:**
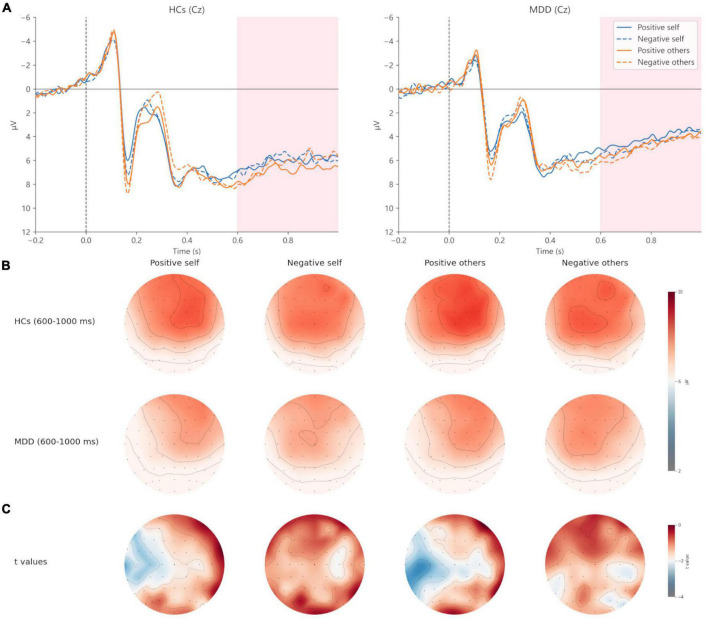
Grand averages **(A)** of LPP (600–1,000 ms) at Cz electrodes under positive self (blue solid lines), negative self (blue dashed lines), positive others (orange solid lines), and negative others (orange dashed lines) condition. Topographic distribution **(B)** of grand averaged LPP within a time window of 600–1,000 ms under four conditions. Topographic distribution of t values for LPP between MDD and HCs **(C)** under four conditions. MDD, major depressive disorder; HCs, healthy controls.

**FIGURE 8:**
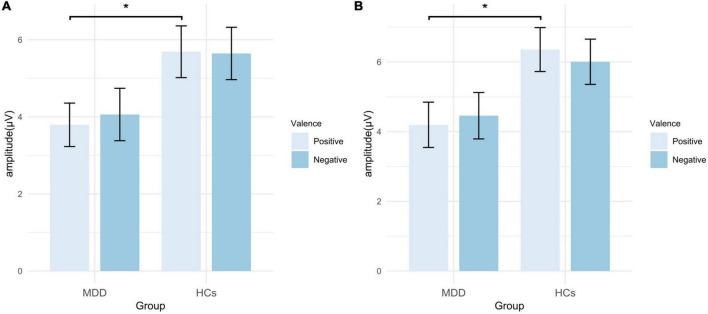
Mean amplitude of LPP in EAST. **(A)** Amplitudes of self-words; **(B)** amplitudes of other-words. MDD, major depressive disorder; HCs, healthy controls; EAST, external affect Simon task; error bars represent the standard error. *p < 0.05.

For the mean amplitude of LPP, the main effect of valence (*F* = 0.153, *p* = 0.697, η^2^p = 0.003) and the interaction were not significant (*F* = 0.302, *p* = 0.585, η^2^p = 0.006). The main effect of group was marginally significant (*F* = 3.965, *p* = 0.052, η^2^p = 0.068). However, the exploratory analysis found that the mean amplitude in the positive self condition was significantly smaller in MDD than that in HCs (*t* = −2.180, *p* = 0.034, Cohen’s *d* = 0.902), while no significant group difference was found in the negative self condition (*t* = −1.640, *p* = 0.107, Cohen’s *d* = 0.754) (Figures 7, 8; Table 3).

#### 3.3.2. Comparison of the ERP differences of other-words between MDD and HCs

For the peak amplitude of N200, valence main effect (*F* = 4.487, *p* = 0.039, η^2^p = 0.077), group main effect (*F* = 5.060, *p* = 0.029, η^2^p = 0.086) and the interaction (*F* = 4.838, *p* = 0.032, η^2^p = 0.082) was significant. *Post hoc* analysis found that in HCs, the absolute value of N200 peak amplitude in the negative others condition was greater than that in the positive others condition (*t* = 2.950, *p* = 0.005, Cohen’s *d* = 0.584). However, no significant results were observed in MDD (*t* = −0.060, *p* = 0.953, Cohen’s *d* = 0.011). Moreover, HCs had a greater absolute value of N200 peak amplitude in the negative others condition than MDD (*t* = 2.833, *p* = 0.006, Cohen’s *d* = 1.404), and no group difference was found in the positive others condition (*t* = −1.543, *p* = 0.129, Cohen’s *d* = 0.809). In terms of the latency of N200, no significant results were found (*p* > 0.05) (Figures 3, 4; Table 3).

For the peak amplitude of P300, the main effect of valence was not significant (*F* = 0.954, *p* = 0.333, η^2^p = 0.017), but the main effect of group (*F* = 5.636, *p* = 0.021, η^2^p = 0.095) and the interaction effect was significant (*F* = 4.940, *p* = 0.030, η^2^p = 0.084). *Post hoc* analysis showed that in HCs, the peak amplitude of positive other-words was greater (*t* = 2.185, *p* = 0.033, Cohen’s *d* = 0.433), while no such difference was found in MDD (*t* = −0.914, *p* = 0.365, Cohen’s *d* = 0.168). In addition, the peak amplitude of positive other-words in MDD was smaller than that in HCs (*t* = −2.906, *p* = 0.005, Cohen’s *d* = 1.602). Regarding the latency of P300, no significant results were found (*p* > 0.05) (Figures 5, 6; Table 3).

For the mean amplitude of LPP, the main effect of valence (*F* = 0.028, *p* = 0.868, η^2^p = 0.001) and the interaction effect were not significant (*F* = 1.331, *p* = 0.254, η^2^p = 0.024), but the main effect of group was significant (*F* = 4.381, *p* = 0.041, η^2^p = 0.075). Exploratory analysis found that the amplitude of positive other-words in MDD was smaller than that in HCs (*t* = −2.367, *p* = 0.022, Cohen’s *d* = 1.100), while no significant difference was found in the negative others condition (*t* = −1.652, *p* = 0.104, Cohen’s *d* = 0.788) (Figures 7, 8; Table 3).

## 4. Discussion

By combining EAST and ERP, the current study investigated the behavioral and neural activity characteristics associated with implicit self-schema and implicit other-schema in MDD. HCs responded faster to positive self-words and positive other-words, while MDD patients did not. Besides, the absolute value of N200 peak amplitude was greater and the P300 peak amplitude was smaller under the negative others condition in HCs compared with MDD patients. Additionally, MDD patients showed smaller LPP amplitudes than HCs in positive self condition and positive others conditions. These results suggested that neural reactivities related to self-schema and other-schema might be altered in MDD.

Compared to negative self-words, HCs responded faster to positive self-words, indicating a closer association between positive attributes and their self-representations. This suggested the existence of positive self-schema in HCs. However, MDD patients did not show this pattern, indicating a lack of positive self-schema. This finding is consistent with some previous studies (10, 16, 19). Nevertheless, Franck et al. (12) used IAT but did not find differences in self-schema between currently depressed patients and HCs. Grundy et al. (26) suggested that this could be due to the confusion between self-schema and other-schema in IAT. Specifically, IAT contains two conditions: positive self condition and negative self condition. The former condition involves pressing the same key (e.g., F) for self-words and positive words, and pressing another key (e.g., J) for other-words and negative words. The latter condition involves pressing the same key (e.g., F) for self-words and negative words, and pressing another key (e.g., J) for other-words and positive words. Therefore, in IAT, the RTs under the positive self condition actually includes the effect of positive self and negative others. Meanwhile, the RTs under negative self condition includes the effect of both negative self and positive others. Since in EAST, object words are presented in different colors and colors are paired with attributes, it can distinguish between self-schema and other-schema effectively.

The RT of positive other-words was smaller than negative other-words in HCs, while no such effect was found in MDD. Moreover, HCs had significantly greater effect of other-EAST than MDD patients. Thus, MDD patients showed a more negative other-schema than HCs. Previous studies have paid less attention to the characteristics of other-schema in MDD and the findings remain controversial. Wu et al. (21) used GNAT and found that HCs responded faster to trials in the negative others condition than to trials in the positive others condition, suggesting the presence of a negative other-schema in HCs. Additionally, Jiang et al. (19) also used GNAT and found that MDD patients had a negative other-schema, but the RT of HCs in the negative others condition was smaller than that of MDD participants, indicating that the other-schema in MDD was more positive than that in HCs. The discrepancies may be due to the other-words used in these studies, which were words that did not refer to a specific object, such as “not me,” “he,” “she,” “others,” etc. Nevertheless, it is worth noting that the other-schema that plays a key role in MDD is the representation of those with whom the individual has intimate relationships, such as parents and partners (29–31). Similar to the present study, the other-words used in Yao et al.’s (16) study were words such as parents and lovers, and they also found that the other-schema in MDD was more negative than that in HCs. However, MDD participants in Yao et al.’s (16) study showed a negative other-schema, whereas in the present study, they only showed a lack of positive other-schema. This difference may relate to the practice effect caused by the increase in the number of trials (32).

Regarding the neural activity patterns of self-schema, although no significant difference was found in N200 and P300, an exploratory analysis of LPP amplitudes showed that MDD patients showed smaller LPP amplitudes than HCs in positive self condition. Since LPP reflects the degree of elaborate processing of stimuli (8, 24), it indicates that MDD patients process positive self-words less elaborately than HCs. Therefore, the above results preliminarily revealed that the abnormal self-schema in MDD patients was only related to the late stage of elaborate processing, which was in line with some existing evidence. For example, Chen et al. (27) using IAT did not find any difference in N200 amplitudes of HCs under the positive and negative self conditions. Allison et al. (22) using SRET found no difference in P300 amplitudes. In addition, Lou et al. (15) also focused on P300 (early LPP) and LPP (late LPP), and significant results were found only for LPP amplitudes. LPP has been considered as a neurophysiological marker of depression (33) and reflects the top-down processes of anterior cingulate cortex (ACC) and prefrontal cortex (PFC) (34). In the process of processing self-related information, ACC involves the identification of motivationally salient stimuli and PFC involves the emotional reappraisal and memory consolidation (34). Compared to HCs, MDD patients showed hyperactivation of PFC and ACC (35, 36), which are associated with self-esteem and depression (36). Therefore, the abnormality of self-schema in MDD may be related to the dysregulated self-cognition and abnormal processing of emotional stimuli based on ACC and PFC. More detailed exploration with functional magnetic resonance imaging (fMRI) will be necessary.

However, some other studies had different findings (6, 19, 25). The inconsistency may be caused by the characteristics of samples and the use of attribute words as the stimuli to induce ERP. Specifically, Allison et al. (22) recruited patients with MDD in remission and Auerbach et al. (6) only included female adolescents with MDD. Though the subjects in Dainer-Best et al.’s (25) study and Jiang et al.’s (19) study were adults with MDD who were currently in an episode, the paradigms they used were SRET and GNAT, respectively, in which positive or negative words were used to induce ERP. Given that MDD patients are characterized by emotion context insensitivity (ECI), referring to the phenomenon that the emotional reactivity of MDD patients is lower than that of HCs when faced with stimuli of different valence (positive adjectives or negative adjectives) (37). Thus, when attribute words are used to induce ERP, it is unclear whether the abnormality of ERP is originated from ECI or the abnormal implicit schemas (24, 38). Unlike IAT, GNAT, and SRET, words used to induce ERP in EAST are object words, which have been proven to activate the implicit attitudes effectively (20, 39–41). Therefore, the results of the present study further clarified that the neural activities related to the implicit self-schema in MDD were mainly reflected in the late processing stage.

Regarding the neural activity patterns of other-schema, the findings were consistent with our hypotheses and behavioral results. For N200 and P300, the analyses showed that in HCs, the absolute value of N200 peak amplitude was greater and the P300 peak amplitude was smaller under the negative others condition. But no such effect was observed in MDD. Since N200 represents response inhibition, error monitoring and mismatch (18), the above results suggested that positive other-words were more consistent with HCs’ implicit attitudes than negative other-words. This indicated that HCs had a positive other-schema, whereas MDD patients lacked it. Since P300 represents the allocation of cognitive and attentional resources (19), the above results showed that HCs allocated more cognitive and attentional resources to positive other-words, while MDD patients did not. Moreover, the absolute value of N200 peak amplitude in the negative others condition and the P300 peak amplitude in the condition of the positive other in HCs were greater than that in MDD, respectively. Thus, in comparison to HCs, MDD participants had more negative implicit attitudes to other-representations and allocated fewer cognitive and attentional resources to positive other-words. For LPP amplitudes, the exploratory analyses revealed that MDD participants had smaller amplitudes than HCs in the positive others condition, suggesting that HCs processed positive other-words more elaborately than MDD patients. These findings provide evidence for theories that emphasize the importance of other-schema, such as cognitive theory, attachment theory and dyadic partner-schema model. Dyadic partner-schema model is proposed by Wilde and Dozois recently, and it demonstrates that the representations of self and others are highly similar, and that negative self-schema and other-schema will reinforce each other in MDD (29). Overall, the above results expand the previous understanding of the neural activity features related to the implicit other-schema in MDD.

Notably, HCs showed larger N200 latency in the positive self condition than the negative self condition, while no such effect was observed in MDD. Also, MDD participants showed greater N200 latency in the negative self condition than HCs. Another study on HCs also suggested that the positive implicit self-schema was associated with the greater N200 latency (21). But other studies did not find similar results. Existing evidence indicates that the N200 latency is influenced by factors such as memory (42) and stimuli evaluation speed (43). Thus, further investigation is necessary to clarify the role of these factors in the current findings. Furthermore, significant group main effects were found in all three ERP components, which may reflect the overall cognitive impairment in MDD (44, 45).

There are some limitations in our study. First, the majority of MDD patients in this study were moderately depressed, and the lack of mild and severe depressed patients might impact the results. Therefore, it is important to include MDD patients with varying degrees of depression to confirm our findings. Second, the relationship between self-schema and other-schema was not the investigated in the current study. Exploring this relationship in future studies may procide further insights into the mechanisms underlying MDD. Third, compared with fMRI, ERP has a higher temporal resolution but a lower spatial resolution. Thus, future studies can use fMRI to comprehensively explore the brain regions or networks involved in implicit schemas of MDD patients. Fourth, although the MDD patients in the current study were not diagnosed with anxiety disorder, they still had mild anxiety symptoms. It is possible that these anxiety symptoms may have influenced our results. Comparing implicit schemas in MDD patients with and without comorbid anxiety disorders in future studies may help to clarify the impact of anxiety on implicit schemas in MDD. Finally, as far as we know, there is currently no study exploring the changes of implicit schemas in MDD following different interventions and their relationships with improvement in depressive symptoms. Investigating these issues may further elucidate the role of implicit schemas in etiology and treatment of MDD.

## 5. Conclusion

Our study explored the neural activity patterns of implicit self-schema and other-schema in MDD by combining ERP and EAST. Both behavioral and ERP indexes showed that MDD participants lacked positive self-schema and positive other-schema. Moreover, implicit other-schema might be related to abnormalities in the early automatic processing stage (i.e., N200 and P300) and the late elaborative processing stage (i.e., LPP), while implicit self-schema might be associated only with abnormalities in the late elaborative processing stage.

## Data availability statement

The raw data supporting the conclusions of this article will be made available by the authors, without undue reservation.

## Ethics statement

The datasets presented in this article are not readily available because the research is still ongoing and the raw data of the current article will be used for further analysis. Requests to access the datasets should be directed to the corresponding author.

## Author contributions

J-YY analyzed the data and drafted the manuscript. J-YY and Z-WZ collected the data. YZ modified the manuscript. S-SS, Y-RW, JT, Y-HP, YW, and W-HJ contributed to the study design. J-YQ was the guarantor, supervised the data collection, statistical analysis, and modified the manuscript. All authors approved the final version of the manuscript.
