# Supplementary material for: Electrophysiological evidence for the characteristics of implicit self-schema and other-schema in patients with major depressive disorder: An event-related potential study
Source: Front Psychiatry. 2023 Apr 11;14:1131275. doi: 10.3389/fpsyt.2023.1131275 (PMC10126260; doi:10.3389/fpsyt.2023.1131275)
Supplement: Supplementary file 1 [file Data_Sheet_1.docx]

Supplementary Material

# Supplementary Data

For self-schema, the main effect of group (F=5.775, p=0.020, η²p=0.097) and valence (F=14.801, p<0.001, η²p=0.215) were significant, but the interaction effect (F=2.271, p=0.138, η²p=0.040) was not significant. However, the exploratory analysis found that HCs responded significantly faster to the positive self-words than to the negative self-words (t=-3.658, p<0.001, Cohen’s d=0.724), whereas no difference was found between negative self-words and positive self-words in MDD patients (t=-1.717, p=0.092, Cohen’s d=0.316). In addition, HCs responded faster to both positive self-words (t=2.733, p=0.008, Cohen’s d=2.360) and negative self-words (t=2.057, p=0.045, Cohen’s d=1.953) than MDD patients. For other-schema, the main effect of group (F=5.989, p=0.018, η²p=0.100), the main effect of valence (F=6.997, p=0.011, η²p=0.115) and the interaction effect (F=5.675, p=0.021, η²p=0.095) were significant. Post hoc analysis found that the RT for positive other-words was significantly greater than that for negative other-words in HCs (t=3.434, p=0.001, Cohen’s d=0.680). And still, no difference was found between negative other-words and positive other-words in MDD patients (t=0.193, p=0.848, Cohen’s d=0.036). Moreover, HCs responded faster to both positive other-words (t=2.764, p=0.008, Cohen’s d=2.677) and negative other-words (t=2.085, p=0.042, Cohen’s d=2.033) than MDD patients. Independent sample t-test was performed on self-EAST effect and other-EAST effect. Though both the self-EAST effect (HCs: 34.05±41.65; MDD: 14.88±51.96) and other-EAST effect (HCs: 32.67±53.60; MDD: 1.71±43.64) of HCs were greater than that of MDD, only the difference of other-EAST effect was significant (other-EAST effect: *t*=2.382, *p*=0.021, Cohen’s *d*= 0.638; self-EAST effect: *t*=1.507, *p*=0.138, Cohen’s *d*= 0.404).

# Supplementary Tables

Supplementary Table 1. Demographic and clinical characteristics of MDD and HCs

|  | HCs (n=26) | MDD (n=30) | *X^2^/t* | *p* |
| --- | --- | --- | --- | --- |
| Age (M±SD) | 28.15±9.01 | 29.47±6.58 | -0.628 | 0.532 |
| Gender (n) |  |  |  |  |
| Male | 8 | 8 |  |  |
| Female | 18 | 22 |  |  |
| Education level (n) |  |  |  |  |
| Middle school | 1 | 3 |  |  |
| College | 25 | 27 |  |  |
| HAMD-17 (M±SD) | 2.08±1.60 | 20.53±3.15 | -28.190 | <0.001 |
| HAMA-14 (M±SD) | 1.00±4.42 | 14.50±4.24 | -15.876 | <0.001 |

MDD, major depressive disorder; HCs, healthy controls.

Supplementary Table 2. RTs of MDD and HCs in EAST (M±SD)

|  |  | Positive | Negative |
| --- | --- | --- | --- |
| HCs | Self-words | 586.99±123.57 | 621.04±131.53 |
|  | Other-words | 592.21±121.85 | 624.88±134.28 |
| MDD | Self-words | 698.01±172.12 | 712.89±191.87 |
|  | Other-words | 720.89±208.36 | 722.61±203.58 |

MDD, major depressive disorder; HCs, healthy controls; RT, reaction time; EAST, External Affect Simon Task; M, mean; SD, standard deviation.
